# Entinostat Enhances Antigen-Specific CD8 T-Cell Response to Immunotherapies in Lung Cancer Models

**DOI:** 10.3390/ph19071034

**Published:** 2026-07-02

**Authors:** Esti Porush, Johnathan Arnon, Baruch Pinchover, Esther Stern, Oz M. Shapira, Didier Jean, Galia Blum, Evalyn Yakobovich, Hanna Wald, Amnon Peled, Zhangmang Wang, Elmehdi Belbaraka, Christian Friese, Thomas Blankenstein, Thomas Kammertoens, Ori Wald

**Affiliations:** 1 Gene Therapy Institute, Hadassah Medical Center, Faculty of Medicine, The Hebrew University of Jerusalem, Jerusalem 9112102, Israel; esti.porush@mail.huji.ac.il (E.P.);; 2 Max Delbrück Center for Molecular Medicine in the Helmholtz Association (MDC), 10117 Berlin, Germany; 3 Institute of Cancer Immunology, Charité Universitätsmedizin Campus Buch, 13125 Berlin, Germany; 4 Sharett Institute of Oncology, Hadassah Medical Center, Faculty of Medicine, The Hebrew University of Jerusalem, Jerusalem 9112001, Israel; 5 Department of General Thoracic Surgery, Division of Cardiothoracic Surgery, Hadassah Medical Center, Faculty of Medicine, The Hebrew University of Jerusalem, Jerusalem 9112102, Israel; 6 INSERM, Université Paris Cité, Sorbonne Université, Centre de Recherche des Cordeliers, F-75006 Paris, France; 7 The Institute for Drug Research, The School of Pharmacy, The Faculty of Medicine, The Hebrew University, Jerusalem 9112102, Israel

**Keywords:** entinostat, non-small cell lung cancer, HDAC inhibition, immune checkpoint inhibitors, TCR-engineered T cells, KRAS

## Abstract

**Background**: Non-small-cell lung cancer (NSCLC) is the leading cause of cancer-related mortality worldwide. Although immune checkpoint inhibitors (ICIs) have significantly improved clinical outcomes, most patients do not respond to treatment or fail to achieve durable responses. Histone deacetylase inhibitors (HDACi) have emerged as promising immunomodulatory agents, with the potential to sensitize tumors to ICIs. We investigated the immunomodulatory effects of entinostat, a class I HDACi, in combination with dual ICI (anti-PD-1 and anti-CTLA-4) as well as with T-cell receptor (TCR) engineered T cells in preclinical NSCLC models. **Methods**: We employed human NSCLC cell lines and the immunogenic KRASG12D/p53-mutant KPN1.1 murine NSCLC cell line. In vitro, we assessed entinostat-induced changes in MHC class I and PD-L1 expression. In addition, we evaluated the effects of entinostat on a KRASG12D-specific TCR. In vivo, therapeutic efficacy and immune modulation were assessed by transplanting KPN1.1 cells subcutaneously and orthotopically into immunocompetent mice, followed by treatment with dual ICI, with or without entinostat. Immune populations in the spleen and blood were subsequently analyzed. **Results**: In vitro, entinostat induced the upregulation of MHC-I and PD-L1 expression in both human and murine NSCLC cell lines. In addition, entinostat treatment significantly enhanced antigen-specific tumor recognition and killing by T cells engineered to express a KRASG12D-specific TCR. In vivo, the addition of entinostat to dual immune checkpoint inhibition showed an incremental trend toward improved tumor growth control. Notably, entinostat plus dual ICI enhanced systemic immune activation, increasing circulating and splenic T-cell populations and significantly expanding both antigen-specific and overall effector CD8^+^ T cells. Consistently, the ex vivo co-culture of splenocytes from KPN1.1-bearing mice with KPN1.1 tumor cells demonstrated enhanced CD8^+^ antigen-specific T-cell recognition. **Conclusions**: In human and murine NSCLC models, entinostat potentiates TCR- and ICI-mediated tumor recognition through tumor-intrinsic and systemic immune modulation. These effects were reflected by increased MHC-I expression, expansion of antigen-specific effector CD8^+^ T cells, and enhanced CD8^+^ T-cell tumor recognition. These findings support a further evaluation of entinostat as a strategy to improve immunotherapy efficacy in NSCLC.

## 1. Introduction

Lung cancer is the second most common malignancy and the leading cause of cancer-related death worldwide, with non-small-cell lung cancer (NSCLC) accounting for approximately 85% of cases [1,2]. Immune checkpoint inhibitors (ICI) have dramatically improved clinical outcomes in metastatic NSCLC and have become the standard of care with or without the addition of chemotherapy [3,4]. In locally advanced resectable NSCLC, and increasingly in investigational approaches for high-risk early-stage disease, neoadjuvant platinum-based chemotherapy and programmed death/ligand-1(PD1/PD-L1) inhibitors are used to promote systemic disease control, enhance pathological response, and subsequently improve disease-free and overall survival [5,6].

However, despite these advances, approximately 20% of patients in early-stage disease and 50% or more of patients in locally advanced or metastatic disease suffer progression and ultimately succumb to their disease, highlighting the need for improved therapeutic strategies [3,4,5,6]. Other T-cell-based immunotherapies, such as autologous tumor-infiltrating lymphocytes and T-cell receptor (TCR) gene therapy, have shown promising early results across several tumor types but have not yet demonstrated meaningful clinical efficacy in NSCLC [7].

One attractive strategy to enhance responsiveness to ICI and TCR therapy in NSCLC is epigenetic modulation by using histone deacetylase inhibitors (HDACi). Mechanistically, HDACi increases histone acetylation and promotes a more relaxed chromatin state, leading to broad transcriptional changes with particular effects on immune-related pathways [8]. HDAC inhibition can promote tumor-intrinsic immunogenicity by increasing MHC class I expression, tumor antigen presentation, and PD-L1 expression, while also reshaping systemic and intratumoral immunity through reducing immunosuppressive myeloid populations and promoting T-cell-permissive immune states [9,10,11,12,13]. Entinostat, a selective class I HDACi, is particularly suited to this approach because of its safe toxicity profile and immunomodulatory activity compared with pan-HDAC inhibitors [8].

Clinical studies with ICI in advanced NSCLC support this rationale but also highlight the limitations of combining HDAC inhibition with single-agent PD-1/PD-L1 blockade in heavily pretreated, ICI-resistant disease. Across studies, entinostat, vorinostat, and mocetinostat combined with single-agent ICI produced modest objective response rates of approximately 9–12% [14]. Nevertheless, the upregulation of MHC-I and proinflammatory tumor microenvironment was observed in post-treatment biopsies, supporting the rationale that HDACi may sensitize tumors to ICI in a treatment-naive setting [10,11]. More recent studies have extended this concept to entinostat in combination with dual ICI, based on the rationale that simultaneous PD-1 and CTLA-4 inhibition may enhance immune priming and overcome exhaustion [13,15]. In malignancies considered resistant to ICI, entinostat combined with nivolumab (anti-PD-1) and ipilimumab (anti-CTLA-4) was well tolerated and produced durable responses in subsets of heavily pretreated patients [15]. Together, these data support the next rational step of testing entinostat with dual ICI in treatment-naive NSCLC, where a less heavily pretreated and potentially less immune-resistant disease context may provide a more favorable setting for HDACi-based immune sensitization [12,13,14,15,16].

In this study, we investigated whether entinostat is suitable for incorporation with anti-PD-1 and anti-CTLA-4, as well as emerging T cell-based antigen-specific targeting strategies. In vitro, we characterized its tumor-intrinsic effects across a panel of NSCLC cell lines, focusing on MHC class I and PD-L1 expression and its ability to enhance antigen-specific recognition and killing by TCR-engineered T cells. In vivo, we used the immunogenic, ICI-responsive KPN1.1 model to evaluate antigen-specific anti-tumor immunity. Using subcutaneous and orthotopic syngeneic models, we assessed how entinostat modulates systemic immune activation and antigen-specific CD8^+^ T-cell responses in the context of anti-PD-1 and anti-CTLA-4 therapy.

## 2. Results

### 2.1. Entinostat Treatment Upregulates Immune-Related Surface Markers

**Figure 1:**
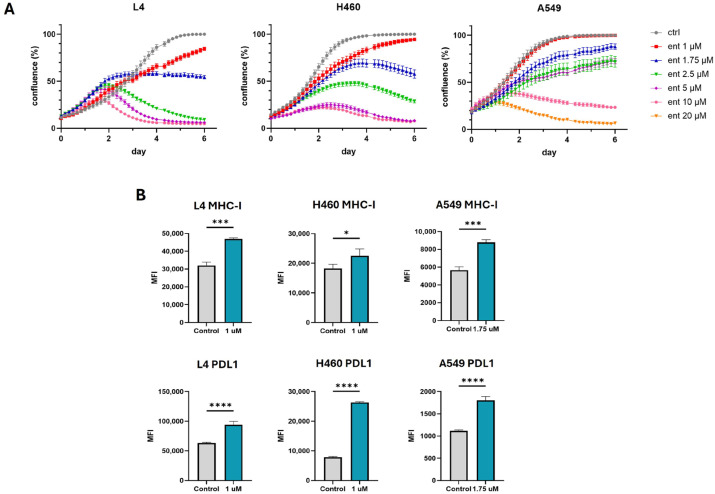
Entinostat upregulates immune-related surface markers in vitro: (**A**) IncuCyte analysis of cell confluence over time in human NSCLC cell lines (L4, H460, A549) following treatment with increasing concentrations of entinostat (ent). (**B**) Flow cytometric analysis of MHC class I and PD-L1 expression in human NSCLC cell lines 72 h after treatment with low concentrations of entinostat. Data are presented as mean ± SEM from biological replicates. Statistical significance was determined using an unpaired two-tailed Student’s *t*-test. * *p* ≤ 0.05; *** *p* ≤ 0.001; **** *p* ≤ 0.0001.

To examine the tumor-intrinsic immunomodulatory effects of entinostat, we assessed its impact on the expression of immune-related molecules in NSCLC cell lines. We used two well-characterized human NSCLC cell lines, A549 and H460, along with the L4 cell line developed in our laboratory as previously described [17]. To identify entinostat concentrations that exert a measurable cellular effect while avoiding marked cytotoxicity, each cell line was treated with a range of entinostat concentrations and monitored longitudinally using the IncuCyte S3 live-cell imaging system. Concentrations that maintained >70% confluence were selected for subsequent analyses. Notably, these doses are equivalent to the doses of entinostat used in previous preclinical studies [10,12]. Under these conditions, entinostat treatment led to the upregulation of MHC class I and PD-L1 expression across all tested cell lines, as determined by flow cytometry, which is consistent with prior reports (Figure 1B).

### 2.2. Entinostat Enhances KRASG12D-Specific TCR-Mediated Tumor Recognition and Killing

TCR gene therapy, i.e., the grafting of tumor antigen specificity onto patients’ T cells by TCR genetic engineering, represents a promising therapeutic approach for ICI-resistant or refractory patients [18]. The success of TCR therapy depends on sufficient expression, processing, and presentation of the target antigen on the MHC-I molecule to the respective engineered CD8^+^ T cells. In a landmark case study, a KRASG12D-specific TCR was successfully used to treat a patient with refractory pancreatic adenocarcinoma (PDAC), providing the first evidence that KRASG12D is immunogenic and can serve as a target for TCR therapy [19]. However, subsequent clinical trials utilizing KRASG12D-specific TCRs have shown only limited efficacy, in part due to the low antigen presentation in KRAS-mutated tumors [20].

**Figure 2:**
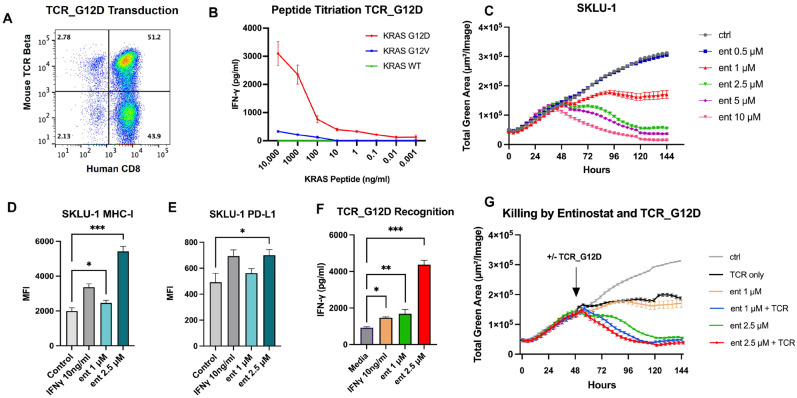
I Enhancing KRASG12D-specific TCR tumor recognition and killing: (**A**) Retroviral transduction of KRASG12D-specific HLA*A11:01 (A11)-restricted TCR (TCR_G12D) in human primary T cells, measured by flow cytometry for CD8 (gated on CD3) and mouse TCR beta constant region, showing 51.2% positively transduced T cells. (**B**) Peptide titration of K562-A11 cells loaded with different concentrations of KRASG12V, KRASG12D, or KRASWT peptide and co-cultured for 24 h with T cells transduced with TCR_G12D, measured by ELISA for IFN-γ. (**C**) IncuCyte analysis of GFP-labeled SKLU-1-A11 cells following treatment with increasing concentrations of entinostat (ent). (**D**–**F**) Flow cytometric analyses of MHC class I and PD-L1 expression in SKLU-1-A11 cells 48 h after treatment with low concentrations of ent or IFN-γ. (**F**) Tumor recognition of SKLU-A11 cells preconditioned for 48 h with ent or IFN-γ and co-cultured for an additional 24 h with T cells transduced with TCR_G12D, measured by ELISA for IFN-γ. (**G**) Tumor killing of SKLU-A11 GFP-labeled cells treated with low doses of ent with or without the addition of T cells transduced with TCR_G12D, measured by GFP readout in the IncuCyte system. For all TCR recognition and killing assays, bulk T-cell product was used at an effector-to -target ratio of 1:1, corresponding to an effective TCR-transduced effector-to-target ratio of approximately 1:2. Data are presented as mean ± SEM from two independent experiments, each performed with technical replicates. Statistical significance was determined using multiple *t*-tests with Holm–Šídák correction. * *p* ≤ 0.05; ** *p* ≤ 0.01; *** *p* ≤ 0.001.

To test our therapeutic approach in this complementary and clinically relevant antigen-specific setting, we investigated whether entinostat could enhance TCR-mediated recognition and killing in KRASG12D models. Previous mass spectrometry and peptide-binding assays reveal that KRASG12D is best presented by HLA-A*11:01 (A11) [21,22]. We therefore focused on a KRASG12D-specific TCR restricted by A11 that was previously isolated from TIL of a colorectal patient, termed here TCR_G12D (NIH; [23]). To establish the system, primary human donor T cells were retrovirally transduced with TCR_G12D. In order to increase expression and prevent mispairing with endogenous TCRs, human TCR constant regions were replaced with murine counterparts. This modification also enabled assessment of transduction efficiency by detection of the murine TCR constant region by flow cytometry, resulting in a transduction rate of 51.2% (Figure 2A). To verify antigen specificity and assess TCR affinity, K562 cells (immortalized myelogenous leukemia cells lacking endogenous MHC-I expression) were retrovirally transduced with A11, loaded with KRASG12D, KRASG12V, or KRAS WT peptide, and co-cultured with T cells expressing TCR_G12D, demonstrating KRASG12D-specific A11-restricted TCR recognition (Figure 2B). Finally, SKLU-1 cells (NSCLC, KRASG12D) were retrovirally transduced to express A11 linked by an IRES element to a GFP reporter, allowing enrichment of A11-positive cells.

To determine the effects of entinostat in this system, GFP-labeled SKLU-1-A11 cells were treated with a range of entinostat concentrations, and GFP readout was monitored over time in the IncuCyte system. Dose-dependent effects similar to those observed in other NSCLC cell lines were observed (Figure 2C). To evaluate the effects of entinostat on antigen presentation and T-cell recognition, SKLU-1-A11 cells were treated with low concentrations of entinostat, as determined above, or IFN-γ as a positive control, leading to the upregulation of MHC-I and PD-L1 in comparison to the untreated controls (Figure 2D,E). Treated cells were then washed and co-cultured for 24 h with T cells expressing TCR_G12D. Both entinostat and IFN-γ preconditioning resulted in a significant increase in recognition of target tumor cells by TCR-transduced T cells relative to untreated controls (Figure 2F). To assess TCR-mediated killing and evaluate the added effect of entinostat, GFP-labeled SKLU-1-A11 cells were treated with low doses of entinostat or left untreated and monitored in the IncuCyte system. After 48 h of treatment, T cells expressing TCR_G12D were added or not added to the IncuCyte culture. Treatment with a combination of low doses of entinostat (1–2.5 uM) together with TCR-engineered T cells resulted in enhanced tumor cell killing, compared with treatment with low doses of entinostat or TCR-transduced T cells alone, which showed only control of tumor growth (Figure 2G).

### 2.3. Entinostat Enhances Functional CD8 T-Cell Responses During Dual ICI Therapy

Alongside its effect on tumor-intrinsic immunity, entinostat has been shown to influence systemic immune responses. To examine these effects in an immunocompetent system that preserves tumor–immune interactions, we utilized the murine KPN1.1 cell line developed by the Joshi laboratory (Yale University) [24]. KPN1.1 cells harbor oncogenic KRASG12D and p53 mutations and were engineered with the NINJA (inversion-inducible joined neoantigen) system, enabling consistent expression of the LCMV-derived immunogenic antigens GP33 and GP66 contained within a GFP loop. These peptides are presented on MHC-I and MHC-II, respectively, and have been shown to elicit CD8^+^ and CD4^+^ T-cell responses [25]. Importantly, KPN1.1 tumors are immunogenic and responsive to dual ICI, making this model suitable for evaluating ICI-enhancing strategies [24]. Notably, antigen-specific T-cell activation in the KPN1.1 model is not mediated by KRASG12D, but rather by the engineered viral-derived GP33/GP66 neoantigens.

**Figure 3:**
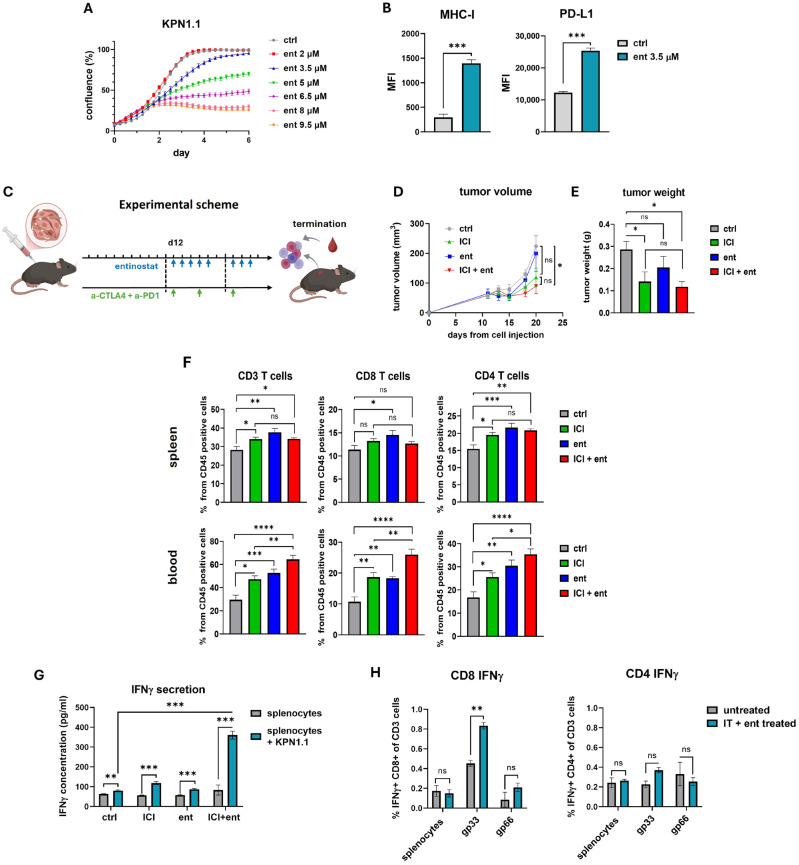
Combined treatment with ICI and entinostat in the KPN1.1 subcutaneous model: (**A**) Longitudinal IncuCyte-based confluence analysis of KPN1.1 cells treated with increasing concentrations of entinostat. (**B**) Flow cytometric analyses of MHC class I and PD-L1 expression in KPN1.1 cells following 72 h treatment with low concentrations of entinostat. (**C**) C57BL/6 mice were subcutaneously injected with KPN1.1 cells and, starting on day 12, treated for 10 days with entinostat, dual ICI (anti–PD-1 and anti–CTLA-4), or the combination (*n* = 8 mice per group). Tumor volume was monitored throughout treatment (**D**), and tumor weight was measured at endpoint (**E**). Immune cell populations in spleen and peripheral blood were analyzed by flow cytometry (**F**). Splenocytes from treated and untreated mice were co-cultured with KPN1.1 cells, and IFN-γ secretion was measured by ELISA (**G**). Additionally, splenocytes were stimulated with NINJA peptides (gp33, gp66) and analyzed for intracellular IFN-γ by ICS (**H**). Data are presented as mean ± SEM. Statistical significance was determined using an unpaired two-tailed Student’s *t*-test (**B**), a repeated-measures ANOVA with correction for multiple comparisons (**D**), one-way ANOVA with correction for multiple comparisons (**E**,**F**), or multiple *t*-tests with Holm–Šídák correction (**G**,**H**). * *p* ≤ 0.05; ** *p* ≤ 0.01; *** *p* ≤ 0.001; **** *p* ≤ 0.0001.

Similarly to the approach used in human tumor cell lines, low entinostat concentrations in KPN1.1 cells were selected based on longitudinal confluence, cell morphology, and LDH cytotoxicity assessment (Figure 3A, Supplementary Figure S1). Under these conditions, entinostat increased MHC-I and PD-L1 expression in KPN1.1 cells (Figure 3B), supporting the use of this model to assess both tumor-intrinsic and systemic immune effects in vivo.

Prior to implantation, GFP expression in KPN1.1 cells was confirmed to verify maintenance of neoantigen expression (Supplementary Figure S2). KPN1.1 cells were then injected subcutaneously into C57BL/6 mice. Twelve days later, mice were treated with entinostat, dual ICI, or their combination (Figure 3C). Treatment with dual ICI showed a trend toward reduced tumor volume over time, whereas the combination of dual ICI with entinostat resulted in a significant reduction compared with the control. However, tumor volume reduction in the combination group was not significantly different from ICI alone. At endpoint, both dual ICI and the combination treatment significantly reduced tumor weight compared with the control, with no significant difference between the ICI-only and combination groups (Figure 3D,E). Despite this modest additional effect on tumor growth, immune profiling of splenic and peripheral blood populations revealed an expansion of T-cell populations in mice treated with either entinostat or entinostat plus dual ICI, including both CD4^+^ and CD8^+^ T-cell subsets (Figure 3F).

To further assess the systemic immune activation elicited by entinostat plus ICI, splenocytes derived from tumor-bearing mice were co-cultured with KPN1.1 cells. Remarkably, only splenocytes derived from mice treated with the combination treatment secreted significantly higher levels of IFNγ upon co-culture with KPN1.1, indicating enhanced systemic anti-tumor immune activation and tumor-specific recognition (Figure 3G).

To define the T-cell subset that produces IFNγ and its antigen specificity, splenocytes from tumor-bearing mice from all treatment groups were stimulated ex vivo with the gp33 or gp66 peptides. Intracellular cytokine staining (ICS) for IFNγ showed that CD8^+^ T cells produced IFNγ upon stimulation with the MHC-I-presented gp33 peptide, whereas no significant increase was observed following stimulation with the MHC-II-presented gp66 peptide. Notably, despite CD4^+^ T-cell expansion in the spleen and peripheral blood (Figure 3F), IFNγ production by CD4^+^ T cells was not significantly altered following stimulation with either peptide (Figure 3H).

### 2.4. Entinostat Promotes Effector T-Cell Expansion During ICI Therapy in an Orthotopic NSCLC Model

To further evaluate the immunomodulatory effect of adding entinostat to dual ICI therapy under physio-immunological and clinically relevant settings, we next transplanted KPN1.1 cells directly into the left lung parenchyma of mice. This approach, which we have previously described extensively, generates a solitary pulmonary nodule surrounded by normal lung parenchyma [26,27]. Hence, it is particularly suitable for testing local and systemic tumor–immune interactions and provides a relevant platform to assess ICI-enhancing strategies.

**Figure 4:**
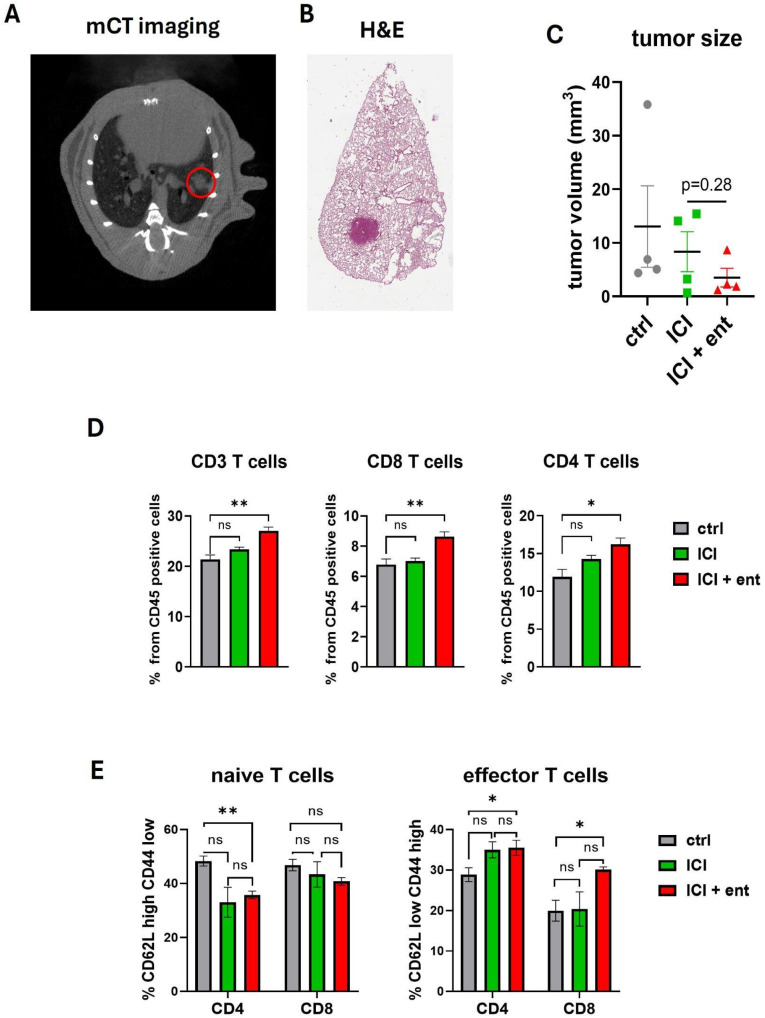
Combined treatment with dual ICI and entinostat in a murine NSCLC orthotopic model: (**A**) Representative CT image of an orthotopic KPN1.1 tumor in the left lung of a mouse. The tumor is indicated by a red circle. (**B**) Corresponding H&E staining of a paraffin-embedded lung section. (**C**) Tumor volume in treated and untreated mice, as evaluated by CT imaging on day 22 after tumor cell implantation (*n* = 4 mice per group). (**D**) Flow cytometric analysis of splenic T-cell populations in tumor-bearing mice following treatment. (**E**) Flow cytometric analysis of splenic T-cell differentiation states. Data are presented as mean ± SEM. Statistical significance was determined using one-way ANOVA (**C**,**D**) or multiple *t*-tests (**E**) with Holm–Šídák correction. * *p* ≤ 0.05; ** *p* ≤ 0.01.

Based on the subcutaneous experiment, in which entinostat alone did not produce a significant tumor control effect, the orthotopic experiment was designed to focus on its potential to enhance dual ICI-associated immune responses rather than on entinostat as monotherapy. Following tumor cell transplantation, treatment with dual ICI, or their combination with entinostat, was initiated on day 11, and the mice were terminated on day 25. Tumor development and volume were assessed by micro computed tomography (mCT) scanning on day 22, and confirmed by H&E staining, which demonstrates a well-defined tumor surrounded by intact lung parenchyma (Figure 4A,B). A non-significant trend for reduction in tumor growth upon addition of entinostat to ICI was observed (Figure 4C). Consistent with our findings in the subcutaneous model, splenic immune population analysis revealed expansion of both CD4^+^ and CD8^+^ T-cell populations following treatment with the ICI plus entinostat combination (Figure 4D). Moreover, immune stainings directed at identifying the activation states of these cells indicated that treatment with ICI plus entinostat reduced the proportion of naïve T cells while increasing effector T-cell populations, with the most prominent effect observed among CD8^+^ T cells (Figure 4E).

## 3. Discussion

In this study, we sought to explore the immunomodulatory effects of entinostat in NSCLC and its potential to support T-cell-based responses to immunotherapies. Previous studies have implicated HDAC inhibition in several aspects of tumor-immune regulation, including reduction in myeloid suppressor cells, remodeling of the tumor microenvironment, and improving immunogenicity through the upregulation of MHC-I and antigen expression [9,10,12,13]. Building on these findings, we focused on T-cell-mediated immunity and antigen-specific tumor recognition, examining both tumor-intrinsic and systemic immune effects of entinostat in the context of T cell-based immunotherapies, including ICI and TCR-engineered T cells.

In vitro, entinostat increased MHC class I expression across human and murine tumor cell lines and importantly improved antigen-specific recognition and killing by a KRASG12D-specific TCR, suggesting enhanced immune visibility. In parallel, entinostat also induced PD-L1 expression, suggesting that specific HDAC inhibition creates a dual phenotype of increased tumor recognition together with checkpoint-mediated responsiveness. This provides a mechanistic rationale for combining entinostat with ICI rather than using HDAC inhibition as monotherapy [10,28].

The effect of entinostat on antigen presentation is particularly relevant for T-cell-based therapies. Downregulation of MHC-I is a well-established mechanism of immune escape across multiple cancer types and may be particularly relevant in KRAS-mutant tumors, where limited presentation of mutant KRAS remains a major barrier to effective TCR-based therapies [9,21,22]. Despite the availability of high-avidity KRAS-specific TCRs with cytotoxic potential, clinical efficacy has remained limited [18,19,23]. In this context, the upregulation of MHC-I following entinostat treatment provided a rationale for testing whether HDAC inhibition could enhance TCR-mediated tumor recognition. Consistent with this rationale, entinostat increased the functional recognition and killing of KRASG12D-expressing tumor cells by KRASG12D-specific TCR-engineered T cells. Thus, combining entinostat or other immunomodulating agents might overcome low MHC-I expression and antigen presentation levels, which have so far hindered clinically meaningful KRAS-targeting TCR therapies. Notably, the increase in TCR-mediated recognition exceeded the degree of MHC-I upregulation, suggesting that additional mechanisms may contribute to the enhanced T-cell response. Further mechanistic studies are needed to determine whether these effects result from an increased presentation of KRASG12D peptide–HLA complexes, broader modulation of the antigen-processing and presentation machinery, or other tumor-intrinsic effects induced by HDAC inhibition.

In vivo, the addition of entinostat to dual ICI was associated with enhanced functional immune activation. Combination treatment increased circulating and splenic T-cell populations and promoted a shift toward an effector phenotype, most prominently within the CD8^+^ T-cell compartment. Moreover, peptide-stimulation experiments demonstrated that the response was not merely a broad expansion of T cells but included a functional antigen-specific CD8^+^ component, as reflected by gp33-induced IFNγ production. By contrast, the observed CD4^+^ T-cell expansion did not translate into detectable antigen-specific CD4^+^ functionality in this assay, as gp66-induced IFNγ production was not significantly enhanced. Importantly, this was demonstrated both in the commonly used s.c. model and in an orthotopic model, which closely mimic the physiological lung tumor microenvironment, thereby strengthening the clinical relevance of our findings [26,27].

Nevertheless, this enhanced immune-effector phenotype was accompanied by only a modest effect on tumor growth, indicating that systemic immune activation did not translate into an equivalent therapeutic impact within the tumor tissue. This disconnect may reflect limited trafficking of activated T cells into the tumor core, insufficient intratumoral engagement, or persistent local immunosuppressive mechanisms within the tumor microenvironment. Effective tumor rejection depends not only on peripheral immune activation, but also on appropriate treatment timing, efficient T-cell trafficking into the tumor, sustained intratumoral engagement, and the ability to overcome local suppressive mechanisms within the TME.

The modest tumor growth effect may also reflect the importance of therapeutic context. Clinical studies of HDAC inhibition with ICI in NSCLC and other cancers [14,29] have largely focused on heavily pretreated or ICI-refractory patients, where established immune resistance may be difficult to reverse. In contrast, the KPN1.1 model is immunogenic, ICI-responsive, and dual checkpoint blockade alone produced a measurable anti-tumor effect, potentially limiting the additional therapeutic window for entinostat [24]. The optimal setting for entinostat-based immune sensitization may therefore lie between these extremes: tumors that are not yet deeply ICI-resistant but in which the anti-tumor immune response remains incomplete and can be further potentiated. This concept may be particularly relevant to the first-line setting, where immune sensitization before extensive therapy-induced resistance could enhance the depth of response.

The treatment sequence may further shape this effect. Since entinostat increased MHC-I and PD-L1 expression, a short entinostat run-in before ICI may allow tumor-cell immune priming before checkpoint blockade is initiated. Such sequential approaches have already been incorporated into clinical studies, including ETCTN-9844, in which entinostat run-in followed by nivolumab (a-PD1) with or without ipilimumab (a-CTLA4) showed preliminary activity in advanced solid tumors, with an ORR of 16% [15]. In the subsequent HER2-negative breast cancer expansion, entinostat combined with nivolumab and ipilimumab achieved an ORR of 25% among evaluable patients, including 40% in triple-negative breast cancer [30]. Future studies in our lab are directed at comparing concurrent and sequential treatment regimens to determine whether a short entinostat preconditioning phase can improve the translation of immune activation into intratumoral engagement and tumor control.

Recent work in KRAS-mutant NSCLC further supports the idea that the therapeutic value of entinostat is highly context-dependent and can extend beyond immune stimulation to synergism with targeted therapies. In particular, in KRAS-mutant NSCLC, responsiveness to ICI is highly dependent on co-occurring mutations, namely STK11/LKB-1 [31], which create an immunosuppressive TME. In KRAS/LKB1-mutant tumors, entinostat enhanced the effect of the MEK inhibitor trametinib by disrupting an HDAC3-dependent tumor-intrinsic resistance program [32]. These findings suggest that entinostat may be particularly effective in tumors with HDAC-regulated vulnerabilities, especially when paired with therapies that expose or target those vulnerabilities. More broadly, they position entinostat as a context-dependent sensitizing agent, whose therapeutic impact may depend on pairing immune modulation with interventions that address additional barriers to tumor control.

Notably, this study has several limitations. First, although entinostat appeared to provide an additional tumor control effect when combined with dual immune checkpoint inhibition, this benefit was modest and did not reach statistical significance in direct comparison with immune checkpoint inhibition alone. Second, the orthotopic experiment was designed as a proof-of-concept study and included a limited number of mice, which may have reduced the statistical power to detect treatment-related differences. In addition, this experiment did not include an entinostat-only treatment arm, limiting our ability to distinguish the independent contribution of entinostat from its combinatorial effect with immunotherapy. Third, given ethical considerations, control and ICI-only mice did not receive vehicle gavage, which may represent a potential confounder when interpreting systemic immune readouts. Finally, only male mice were used in this study, mandating future validation across both sexes.

In summary, our findings support entinostat as a potential immune-sensitizing strategy in NSCLC, with the capacity to increase tumor-cell immune visibility and strengthen antigen-specific CD8^+^ T-cell responses in the context of both checkpoint blockade and TCR-engineered T-cell therapy. The modest tumor growth effect observed in this study points to the need to better define the conditions under which entinostat-induced immune modulation is translated into effective tumor control. These may include optimization of the treatment sequence, evaluation in therapeutic settings with an incomplete but still modifiable immune response, and combination with complementary approaches that enhance tumor susceptibility to T-cell–mediated attack. This framework supports further investigations of entinostat-based strategies as part of rationally designed T-cell-based immunotherapy combinations in NSCLC.

## 4. Materials and Methods

### 4.1. Cell Lines

Human NSCLC cell lines A549, H460, and SKLU-1 were obtained from ATCC. The L4 primary NSCLC cell line was established in our laboratory, as previously described [27]. The murine KPN1.1 cell line, stably engineered with the NINJA system, was kindly provided by the Joshi laboratory (Yale University). Human cell lines were cultured in RPMI 1640 or DMEM, and KPN1.1 cells in DMEM, both supplemented with 10% fetal bovine serum, L-glutamine, penicillin–streptomycin, and sodium pyruvate. Cells were maintained at 37 °C in a humidified atmosphere with 5% CO_2_ and were routinely tested for mycoplasma contamination.

### 4.2. Peptide

KRAS peptides were synthesized by GenScript (Piscataway, NJ, USA)—KRAS G12V (VVGAVGVGK), KRAS G12D (VVGADGVGK), and KRAS WT (VVGAGGVGK) LCMV peptides GP33-41 (KAVYNFATM) and GP61-80 (GLKGPDIYKGVYQFKSVEFD) were purchased as synthetic peptides from MedChemExpress (MCE, Monmouth Junction, NJ, USA).

### 4.3. Incucyte Assay

Cells were seeded in 96-well plates at a density of 3000–10,000 cells per well for human cell lines and 750 cells per well for KPN1.1 cells. After 24 h, cells were treated with increasing concentrations of entinostat (MS-275; Selleck Chemicals, Cat# S1053, Houston, TX, USA) that were diluted using a DMSO stock solution. Cell confluence or the GFP signal (for labeled cells) was monitored every 4 h using the IncuCyte live-cell imaging system (Sartorius, Göttingen, Germany).

#### 4.3.1. Entinostat Treatment and Flow Cytometry Analysis

Cells were treated with low concentrations of entinostat, as determined by IncuCyte analysis (1–3.5 µM). After 48 or 72 h, cells were harvested and analyzed for MHC-I and PD-L1 expression by flow cytometry. Cells were stained for 30 min at 4 °C in PBS using antibodies purchased from BioLegend (San Diego, CA, USA) at a 1:100 dilution, as detailed in Supplementary Table S1. Unstained controls were included in each experiment, and compensation was performed using single-stained controls. Samples were acquired on a CytoFLEX flow cytometer (Beckman Coulter, Brea, CA, USA), and data were analyzed using CytExpert software (Beckman Coulter, Brea, CA, USA).

#### 4.3.2. Mice

C57BL/6J mice were purchased from Harlan; 7–10-week-old male mice were used for all studies.

### 4.4. Tumor Models

Prior to implantation in both subcutaneous and orthotopic tumor experiments, GFP expression in KPN1.1 cells was verified by flow cytometry, and only cultures with >95% GFP-positive cells were used.

For the subcutaneous tumor model, KPN1.1 cells (200 K cells/mouse) were injected into the right flank of C57BL/6J mice, with at least six mice per group. Tumor size was routinely monitored using a caliper, and tumor volume was calculated according to the formula V = 0.5 × length × width^2^. At the experimental endpoint, mice were euthanized, tumors were excised and weighed, and spleens and peripheral blood were harvested for further analysis.

Orthotopic lung tumors were established by intrapulmonary injection, as previously described [26,27]. Briefly, KPN1.1 cells were resuspended in DMEM, mixed 1:1 with Matrigel, and kept on ice until injection. Mice were anesthetized with ketamine/xylazine, and 2 µL of the cell/Matrigel suspension, corresponding to 5000 cells per mouse, was injected into the left lung parenchyma between the fourth and fifth ribs using a Hamilton syringe fitted with a 31-gauge needle. Following injection, the incision was closed, and mice received analgesia and were allowed to recover. At the experimental endpoint, mice were euthanized by ketamine/xylazine overdose. The chest cavity was exposed, and lungs were perfused through the trachea with PBS. Lungs were then harvested, fixed, embedded in paraffin, sectioned, and stained with hematoxylin and eosin (H&E) for histological assessment.

### 4.5. Entinostat and Immune Checkpoint Inhibitor Treatment

For the subcutaneous tumor experiments, mice were randomized on day 12 after tumor inoculation into four treatment groups: control, entinostat, ICI, or combination treatment with entinostat and ICI. ICI treatment consisted of anti-PD-1 (clone RMP1-14; Bio X Cell, Lebanon, NH, USA) and anti-CTLA-4-blocking antibodies (clone 9D9; Bio X Cell, Lebanon, NH, USA), each administered intraperitoneally (i.p.) at 200 µg per dose twice weekly for a total of three doses. Entinostat was prepared from a DMSO stock solution, formulated in 5% DMSO, 40% PEG 300, 5% Tween 80, and 50% saline, and administered by oral gavage (p.o.) at 7.5 mg/kg five times weekly for a total of eight doses over the 10-day treatment period. The dose and oral gavage regimen were based on previous preclinical studies using comparable entinostat dosing schedules [12,13]. Control and ICI-only mice did not receive vehicle gavage to avoid unnecessary repeated oral gavage in accordance with ethical considerations. Body weight was monitored throughout treatment and remained stable, and no overt signs of treatment-related toxicity were observed. Mice were euthanized after 10 days of treatment for endpoint analysis.

For the orthotopic lung tumor experiments, treatment was initiated on day 11 after tumor inoculation and continued for two weeks using the same entinostat and ICI doses and administration frequencies described above. Micro-CT imaging was performed on day 22 for tumor volume assessment, and mice were euthanized on day 25 for endpoint immune and histological analyses.

#### 4.5.1. Micro-CT

Micro-CT scans were performed using MILabs U-CT Low-Dose Ultra-High-Resolution Micro-CT System (MILabs, Houten, The Netherlands) with specific parameters: Ultra-Focus magnification, single energy source, and normal scan mode (360° rotation, 60 kV, 0.16 mA, 8 projections per step with a 0.800° step angle, 50 ms exposure time, and 1 × 1 binning). A fixed 100 µm aluminum filter and a standard aluminum 400 µm filter were used. Three-dimensional reconstruction of the Micro-CT using a reconstructed voxel size of 40 µV with Hann and Gaussian (80 micron) filters was generated.

#### 4.5.2. Volumetric Tumor Analyses

Three-dimensional (3D) tumor segmentation and quantitative computed tomography (CT) image analyses were performed using Imalytics Preclinical software (version 3.1.1.8) (Aachen, Germany). Target volumes of interest corresponding to the tumor masses were identified and designated as distinct classes within the software workspace. Volumetric delineation of the tumor boundaries was executed using the Scribble tool within Imalytics Preclinical software. To ensure rigorous 3D spatial coverage, tumor borders were dynamically traced while scrolling through the imaging datasets across both the axial and coronal anatomical planes. A minimum of 3 individual segmentation slices were defined across the tumor volume (mm^3^) to capture its morphological heterogeneity. The Scribble tool was configured with the following standardized operational parameters: 10-pixel gap between foreground and background regions, 5-pixel scribble thickness, and auto and double modes. To maintain the raw spatial characteristics and spatial resolution of the primary segmentations, no post-processing smoothing filters or morphological alterations were applied to the finalized tumor mask.

### 4.6. Preparation of Cell Suspensions for Flow Cytometry Analysis

Peripheral blood was collected into heparin-containing tubes. Spleens were harvested and mechanically dissociated through a 70 µm cell strainer to generate single-cell suspensions. Peripheral blood and spleen cell suspensions were then treated with erythrocyte lysis buffer containing 155 mM NH_4_Cl, 10 mM KHCO_3_, and 0.1 mM Na_2_EDTA, pH 7.4. Cells were washed and stained for flow cytometry analysis, as described above, using the immune profiling antibody panel detailed in Supplementary Table S2. Immune cell populations were defined according to marker expression within the corresponding parent populations.

### 4.7. Co-Culture and ELISA Assay

For co-culture assays, spleens from treated and untreated KPN1.1 tumor-bearing mice were collected, and splenocytes were isolated as described above. The number of viable splenocytes in each sample was determined by trypan blue staining. Furthermore, 24 h prior to the experiment, 3 × 10^4^ KPN1.1 cells per well were plated in flat-bottom, 24-well plates (Corning, New York, NY, USA). Next, the medium was changed to fresh medium and splenocytes were added in a 1:40 ratio (1.2 × 10^6^ splenocytes per well). Additionally, 48 h following the co-culturing of tumor cells and splenocytes, the medium was collected for ELISA assay. IFN-γ levels in the culture medium were quantified using ELISA (DuoSet Mouse IFN-gamma immunoassay, R&D Systems, Minneapolis, MN, USA) according to the manufacturer’s instructions.

To verify the TCR_G12D peptide titration assay, K562-A11 cells were loaded with KRASG12D, KRASG12V, or KRAS WT peptide at different concentrations. After 90 min, cells were washed twice with PBS, co-cultured with T cells expressing TCR_G12D. After an additional 24 h, supernatant was collected, and IFN-γ was measured by ELISA.

For co-culture of TCR-transduced T cells with SKLU-1 expressing A11, cells were seeded in a 6-well plate at 3 × 10^5^ cells per well and treated with Entinostat (1–2.5 μM), IFN-γ (10–50 ng/mL) or left untreated. After 48 h, cells were washed twice with PBS and seeded on a 96-well plate at 2 × 10^4^ cells per well. Two hours later, T cells expressing TCR_G12D were added to the culture, and after an additional 24 h, supernatant was collected, and IFN-γ was measured by ELISA as specified above.

For all TCR_G12D recognition and killing assays (including IncuCyte), bulk T-cell products were co-cultured with target cells at an effector-to-target ratio of 1:1. Based on the observed transduction efficiency of 51.2%, the resulting ratio of TCR-transduced effector cells to target cells was approximately 1:2.

#### Intracellular Cytokine Staining

For intracellular cytokine staining (ICS), splenocytes isolated from treated and untreated KPN1.1 tumor-bearing mice were stimulated ex vivo with gp33 or gp66 peptides at a final concentration of 5 µg/mL for 18 h. Brefeldin A was added during the final 5 h of stimulation at a final concentration of 10 µg/mL to block cytokine secretion. Cells were then stained for surface markers, including CD3, CD4, and CD8, fixed and permeabilized using the eBioscience™ Transcription Factor Staining Buffer Set, and stained intracellularly for IFN-γ. Samples were acquired by flow cytometry as described above, and IFN-γ production was analyzed within the CD8^+^ and CD4^+^ T-cell populations.

### 4.8. Tcr and A11-Gfp Retroviral Transduction

TCR_G12D α- and β-chain sequences containing murine constant regions were retrieved from patent WO 2021/163434 A1, synthesized by GeneArt (Thermo Fisher Scientific, Waltham, MA, USA), and subcloned into the MP71-PRE retroviral expression vector. The MP71 vectors containing TCR_G12D constructs were then transfected into the viral packaging line 293GP-GLV cells or envelope-encoding plasmid RD114 cells using Lipofectamine 2000 reagent (Thermo Fisher Scientific, Waltham, MA, USA). The supernatant containing the viral particles was harvested 24 and 48 h after transfection. In parallel, human primary donor T cells were thawed and activated in human T-cell media for 48 h with anti-human CD3/CD28 antibodies and 200 U/mL of human IL2 prior to viral infection. Two transductions were performed for each experiment: The first transduction was carried out by spinoculation of the viral supernatant with the T cells, at 800× *g* for 90 min and 32 °C. The second transduction was carried out on the following day using the 48 h viral supernatant attached to Retronectin-coated plates (Takara, Shiga, Japan) by centrifugation at 3200× *g* for 90 min at 4 °C, and adding the cells onto the viral-coated plates and spinoculation for an additional 30 min at 32 °C. TCR-transduced T cells were kept at 200 U/mL IL2 for 10 days and at 20 U/mL IL2 for 3 additional days before use. TCR transduction efficacy was confirmed by using mouse TCRβ antibody 8 to 16 days after the day of stimulation.

For the generation of tumor target cells, the HLA-A*11:01 coding sequence was retrieved from the IPD-IMGT/HLA database, codon-optimized for mammalian expression, and synthesized by GeneArt. A11 inserts were cloned into the MP71 retroviral vector upstream of an IRES-GFP reporter cassette. Tumor cells were maintained in RPMI medium and retrovirally transduced using the same protocol described above. GFP expression was used to assess transduction efficiency and to enrich cell populations with >90% GFP positivity.

#### 4.8.1. Statistics

Statistical analyses were performed using GraphPad Prism software (Boston, MA, USA). For comparisons between two groups, statistical significance was determined using an unpaired two-tailed Student’s *t*-test. For comparisons among multiple groups, one-way ANOVA was used, followed by Holm–Šídák correction for multiple comparisons. Longitudinal tumor growth curves were analyzed using repeated-measures ANOVA with Holm–Šídák correction for multiple comparisons. Multiple pairwise comparisons were analyzed using multiple *t*-tests with Holm–Šídák correction. A *p*-value < 0.05 was considered statistically significant. Unless otherwise indicated, all in vitro experiments were performed with at least three technical replicates.

#### 4.8.2. Study Approval

The Animal Care and Use Committee of The Hebrew University approved all experiments.

## Data Availability

The original contributions presented in the study are included in the article and Supplementary Materials, further inquiries can be directed to the corresponding authors.

## Supplementary Materials

The following supporting information can be downloaded at: https://www.mdpi.com/article/10.3390/ph19071034/s1, Figure S1: Morphology and LDH release following entinostat treatment in KPN1.1 cells; Figure S2: Verification of NINJA expression in KPN1.1 cells; Table S1: Flow-cytometry antibodies; Table S2: Panel of antibodies.
