# Supplementary material for: Entinostat Enhances Antigen-Specific CD8 T-Cell Response to Immunotherapies in Lung Cancer Models
Source: Pharmaceuticals (Basel). 2026 Jul 2;19(7):1034. doi: 10.3390/ph19071034 (PMC13416286; doi:10.3390/ph19071034)
Supplement: Supplementary file 1 [file pharmaceuticals-19-01034-s001.zip › pharmaceuticals-4380014-supplementary.pdf]

Supplementary data

application	marker	Supplier	Clone	Fluorophore
**MHC-I**	anti human HLA-A,B,C	Biolegend	W6/32	PE
	anti mouse H-2Kb/H-2Db	Biolegend	28-8-6	APC
**PD-L1**	anti human CD274	Biolegend	29E.2A3	APC
	anti mouse CD274	Biolegend	MIH5	PE
**hematopoietic cells**	anti mouse CD45	Biolegend	30-F11	FITC
**T cells**	anti mouse CD3e	Biolegend	145-2c11	PE/Cy5
	anti mouse CD8a	Biolegend	53-6.7	PE
	anti mouse CD4	Biolegend	GK1.5	APC
	anti mouse CD4	Biolegend	RM4-5	FITC
**T cells activation states**	anti mouse CD44	Biolegend	IM7	APC/Cy7
	anti mouse CD62L	Biolegend	MEL-14	FITC
**IFN-γ ICS**	anti mouse IFN-γ	Biolegend	XMG1.2	APC

Table S1: Flow-cytometry antibodies

Cell population	FITC	PE	PE/CY5	APC	APC/Cy7
**T cells**	CD45	CD8	CD3	CD4	
**T cells activation states**	CD62L	CD8	CD3	CD4	CD44
**IFN-γ ICS**	CD4	CD8	CD3	IFNγ	

Table S2: Panel of antibodies

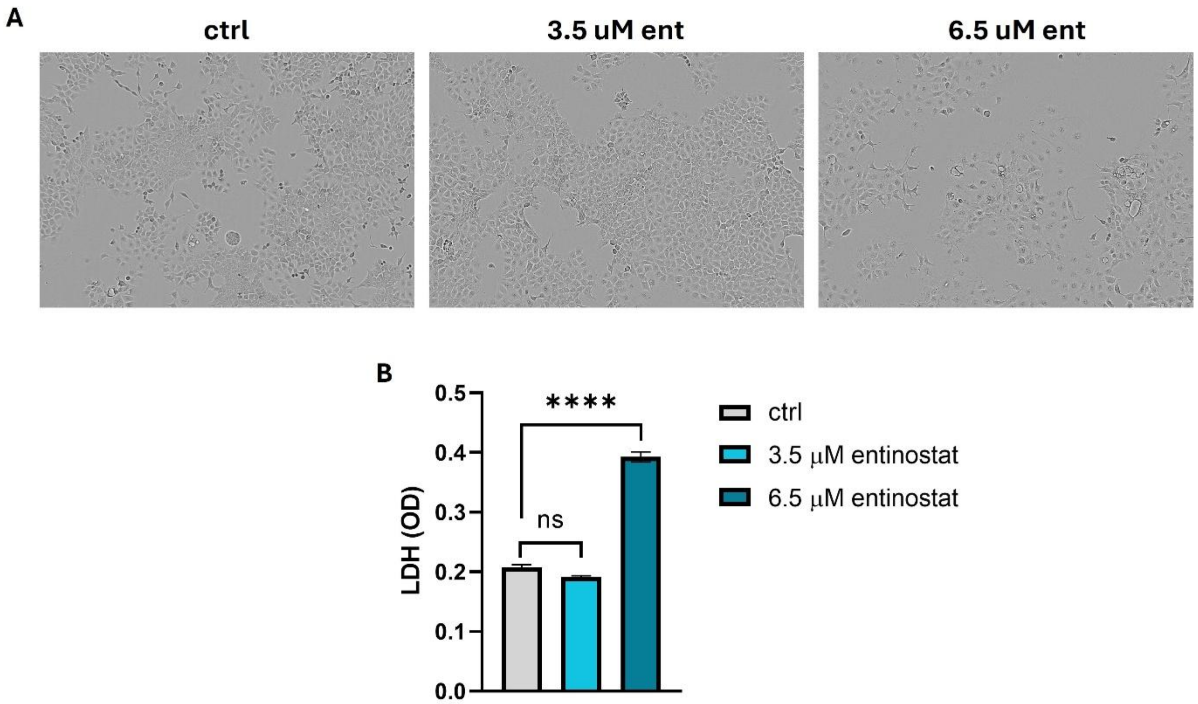

Figure S1: Morphology and LDH release following entinostat treatment in KPN1.1 cells. KPN1.1 cells were treated with 3.5 or 6.5 μM entinostat for 48 h. Representative phase-contrast images show preserved cellular morphology following 3.5 μM entinostat treatment, whereas 6.5 μM entinostat was associated with marked morphological deterioration. LDH release into the culture medium was measured by absorbance and is presented as optical density (OD). Treatment with 3.5 μM entinostat did not significantly increase LDH release compared with untreated control, whereas 6.5 μM entinostat induced a significant increase in LDH signal. Data are presented as mean ± SEM. Statistical significance was determined by one-way ANOVA with multiple-comparisons correction. ns, not significant; ****p < 0.0001.

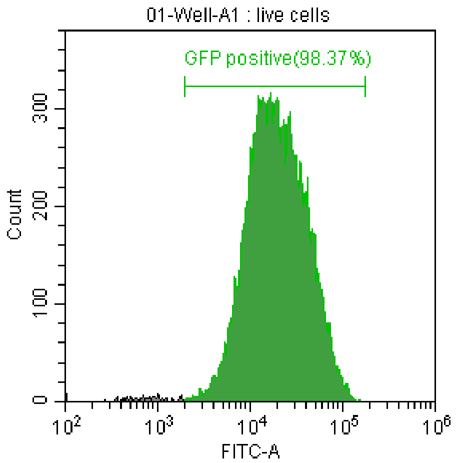

Figure S2: Verification of NINJA expression in KPN1.1 cells. Flow cytometric analysis of GFP expression in live KPN1.1 cells prior to implantation. GFP positivity was used as an indicator of NINJA construct expression, demonstrating that the vast majority of cells expressed the NINJA system, with >95% GFP-positive cells.
